# Nalbuphine Potentiates Reversal of Fentanyl Overdose by Naloxone

**DOI:** 10.3390/ph17070866

**Published:** 2024-07-02

**Authors:** Mihai Cernea, Georgiy Nikonov, Janna Ataiants, Cristina Ştefănuţ, John Abernethy, Michael Voronkov

**Affiliations:** 1 Department of Pharmacology, Faculty of Veterinary Medicine, University of Agricultural Sciences and Veterinary Medicine, 400372 Cluj-Napoca, Romania; mihai.cernea@usamvcluj.ro (M.C.);; 2 Κappa Pharmaceuticals LLC, Alachua, FL 32615, USA; 3 Dornsife School of Public Health, Drexel University, Philadelphia, PA 19104, USA; 4 Serodopa Therapeutics Inc., Gainesville, FL 32601, USA

**Keywords:** fentanyl, overdose reversal, naloxone, nalbuphine, withdrawal

## Abstract

Developing an effective antidote for fentanyl-induced overdose to achieve timely reversal is an unmet public health need. Previously, we found that naloxone derivative NX90 with mild κ-opioid agonistic properties was three-fold more effective than the parent naloxone in reversing a fentanyl overdose in rats. To investigate whether κ-agonistic properties could indeed augment the robustness of overdose reversal, we evaluated a κ-agonist/µ-antagonist nalbuphine (NB) as well as its combinations with naloxone (NX) in a fentanyl overdose model in rodents. An administration of either NB or NX as single agents at 0.1 mg/kg doses produced a full recovery in 90 ± 9.9 min and 11.4 ± 2.7 min, respectively. A higher dose of NX at 0.2 mg/kg reversed an overdose within 4.8 ± 1.0 min. In contrast to that, the coadministration of NB and NX at 0.1 mg/kg each produced a synergistic effect, with overdose reversal in 3.4 ± 0.2 min. The coadministration of NX and NB at sub-therapeutic doses of 0.05 mg/kg each was also 1.2-fold more effective than NX at 0.2 mg/kg. We further found that co-administration of NB at different doses (0.025, 0.05, 0.1 mg/kg) and ratios (1:4 and 1:1) with NX had differential effects on overdose reversal, cardiorespiratory liabilities, and analgesia.

## 1. Introduction

**Figure 1:**
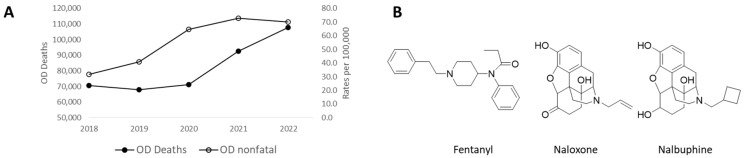
(**A**) Total number of deaths from overdose and rates of non-fatal opioid overdose per 100,000 population in the US (2018–2022). (**B**) Chemical structures of fentanyl, naloxone, and nalbuphine.

Since 2013, there has been a dramatic increase in synthetic opioid-related deaths, primarily due to high-potency opioids such as fentanyl and its analogs. The anomalous pharmacology of fentanyl—including rapid onset of action and reduced sensitivity to reversal by naloxone [1]—has contributed to approximately 68% of the reported 107,081 drug overdose (OD) deaths attributed to synthetic opioids in the United States in 2022 alone (Figure 1) [2]. Apart from mechanical respiratory failure [3,4], the main risk of death from a fentanyl overdose comes from respiratory depression [5]. As the number of non-fatal opioid overdoses reached 69.6 per 100,000 population from 2018 to 2022 [6], the risk burden of the resulting hypoxic injury to the brain that could lead to long term disability [7] may play out in many years to come. Therefore, there is an unmet public health need to find a fentanyl-driven overdose reversal agent.

Naloxone has been the gold standard for reversing an opioid overdose [8]. Since overdose is an adverse effect of an opioid receptor activation, the pharmacological role of naloxone as an antagonist is to outcompete the other opioids for the receptor. However, the increasing death toll from overdoses induced by non-medical fentanyl, a highly potent opioid, requires expeditious delivery of naloxone at higher/repeated doses [9,10], pushing the limits of the intervention safety [11]. Yet, the administration of naloxone to overdose victims has its own liabilities.

Firstly, naloxone precipitates severe withdrawal sickness [12,13]. While spontaneous opioid withdrawal is a debilitating and, in some instances, life-threatening condition [14,15], the severity of naloxone-induced withdrawal was reported, at least in animal studies, to be comparable or greater [16]. Withdrawal syndrome requires active management of symptoms, which may include pain medications for myalgia and medicines for cardiovascular and gastrointestinal side effects [15]. Furthermore, it is well documented that, in some instances, naloxone-induced withdrawal forces patients to self-medicate with street drugs to counter the effects of naloxone [17]. Such practices contribute to the significant rate of post-rescue deaths immediately following hospital discharge [18]. Moreover, hyperalgesia and lower pain tolerance during the opioid withdrawal are prominently associated with increased relapse rates [19].

Secondly, naloxone administration is associated with catecholamine release [20], which is thought to be involved in cardiovascular stimulation [21,22], the most prevalent side effects in overdose patients. Finally, studies have reported that naloxone may not effectively mitigate [23] and sometimes even contributes [24] to fentanyl’s noradrenergic and cholinergic effects (e.g., mechanical failure of respiration or wooden chest syndrome), which are rapid and distinct from respiratory depression seen with morphine-derived alkaloids. Therefore, the development of a new fentanyl overdose reversal agent should strive to be not only more effective than naloxone but also have better withdrawal and safety profiles.

Toward that goal, we recently have shown that the naloxone derivative NX90, with a significantly more lipophilic profile, was more effective in a fentanyl overdose rat model [25]. Unlike naloxone, however, NX90 has mild κ-agonistic properties that raise the question of whether κ-agonism can also augment overdose reversal. Previous reports by others and us have suggested that the activation of κ-opioid receptors may play an important role in regulating the severity of withdrawal [26,27,28]. Furthermore, the interactive effects of µ- and k-agonists on respiratory function in conscious rats have also been reported [29].

In this report, we evaluated a clinically available κ-agonist/µ-antagonist nalbuphine (NB) as well as its various combinations with naloxone (NX) in an overdose reversal model. To the best of our knowledge, this is the first study that demonstrates the role of κ-agonism in a fentanyl-driven overdose rat model.

## 2. Results and Discussion

### 2.1. Evaluation of the Efficacy of NX, NB, and the Combination in a Fentanyl-Induced Overdose Model

**Figure 2:**
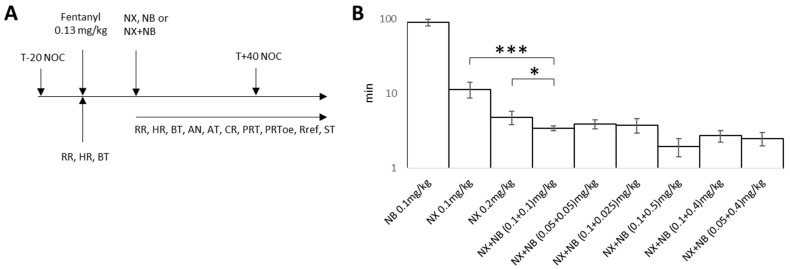
(**A**) Design of the experiment: vitals and reflexes monitored: RR—respiratory rate, HR—heart rate, BT—body temperature, AN—alertness (if the rat responded to acoustic and painful stimuli at the skin level), AT—astasia (if the rat showed locomotor imbalance or stopped moving), CR—corneal reflex (closure of eyelids in response to irritation of the cornea by touching with a sterile cotton applicator), PRT—pinch reflex tail (tail flick when pinching the tail), PRToe—pinch reflex toe (toe flinch when pinching the toe), Rref—righting reflex (the lack of the righting reflex from the decubitus position), ST—sternal recumbency (the rat has no tendency to rise from the sternal recumbency position, in a support position on the 4 limbs). T-20 NOC—nociception pretest 20 min prior to fentanyl administration, T + 40 nociception test 40 min after fentanyl administration. (**B**) The Bonferroni test was used to compare differences of mean time to all reflexes restored (* *p* < 0.05; *** *p* < 0.005).

Using the optimal fentanyl dose from our previous studies [25] that results in overdose but can be reversed with naloxone, sedation/analgesia was induced in rats, and they were monitored individually for respiratory rate (RR), heart rate (HR), and body temperature (BT), as well as eight basic reflexes, including nociception (Figure 2A).

After achieving an overdose (RR ≤ 25%, HR ≤ 80%, no CR), one of the following was administered: NX, NB, or NX + NB combination. As previously published, we qualified an overdose reversal as full restoration of all monitored reflexes and RR to at least 67% of the respiratory rate [25]. The mean time to overdose reversal for each treatment group is shown in Figure 2B. We found that NB (0.1 mg/kg) as a single agent was less effective in reversing a fentanyl-induced overdose compared to NX at both tested doses (0.1 and 0.2 mg/kg), with mean overdose reversal times of 90 ± 9.9, 11.4 ± 2.7, and 4.8 ± 1.0 min, respectively.

However, we observed a statistically significant synergistic effect on overdose reversal when a combination of NX + NB was administered (Figure 2B). Specifically, when therapeutic NX (0.1 mg/kg) was supplemented with an equal dose of NB, a 3.35-fold improvement in mean reversal times was observed. Even compared to the doubled dose of NX (0.2 mg/kg), the combination of NX + NB (at 0.1 mg/kg each) demonstrated a 1.4-fold improvement in mean OD recovery time.

Remarkably, supplementing the therapeutic dose of NX (0.1 mg/kg) with the smallest dose of NB (0.025 mg/kg) produced a three-fold improvement in mean recovery time (3.75 ± 0.8 min). Furthermore, a sub-therapeutic dose of NX (0.05 mg/kg) when supplemented by an equal dose of NB (0.05 mg/kg) was more effective than any tested dose of NX alone with the reversal time of 3.9 ± 0.6 min. Therefore, the observed synergistic effect on overdose reversal cannot be explained simply by additive µ-antagonism from NB, and it is likely that κ-opioid receptors are playing a role.

If that is correct, why did the administration of NB (κ-agonistic and µ-antagonistic) as a single agent fail to improve full recovery times? It requires further studies, but one explanation is that in presence of NB there might be a redistribution of NX from κ-opioid to µ-opioid receptors. Indeed, NB affinity to κ-opioid receptors is about six-fold higher [30] than that of NX, and at the molecular level, NB outcompetes NX for binding to κ-opioid receptors, thus freeing more NX molecules for binding to µ-opioid receptors. Such a boost in µ-antagonistic activity would not be available when NB is administered alone.

Interestingly, a well-documented coadministration of NX and NB also boosts analgesic properties of NB in humans [31,32]. In fact, it was shown that the observed potentiation depends on NB/NX ratios more than specific doses of each agent [31], suggesting that a redistribution between k-opioid and µ-opioid receptors might also be pharmacodynamically relevant.

One would expect that the co-administration of NB would result in lower alertness, a common side effect [33] that could be detrimental to the effective overdose reversal. In fact, the alertness in the NB-only-treated group was the last or next to the last reflex to recover. However, in the combination with NX, even at the highest dose of NB (0.5 mg/kg) the alertness was not reduced compared to NX-only-treated groups.

### 2.2. Evaluation of Respiratory and Cardiovascular Liability of NX, NB, and the Combination in a Fentanyl-Induced Overdose Model

**Figure 3:**
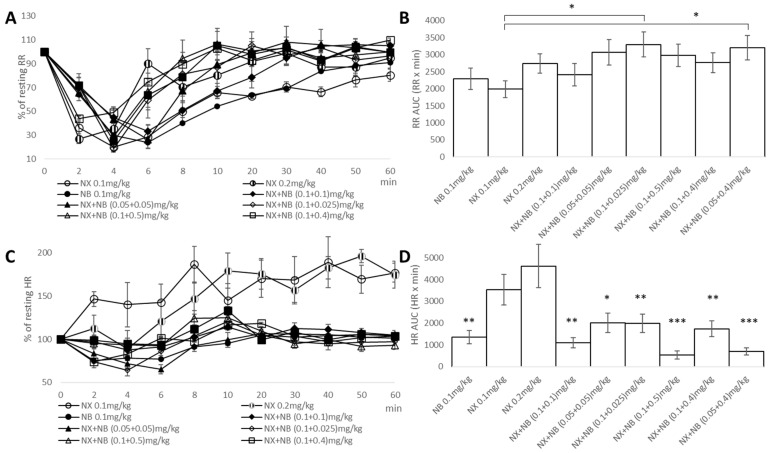

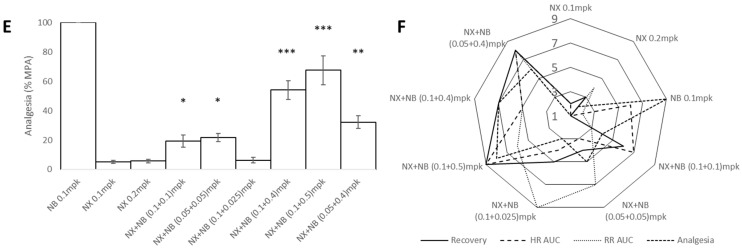
(**A**) Mean heart rate (RR) as a percentage of the resting rate for all treated groups. (**B**) Cumulative measure of respiratory activity for all treated groups (* *p* < 0.05). (**C**) Mean heart rate (HR) as a percentage of the resting rate for all treated groups. (**D**) Cumulative measure of heart rate for all treated groups. The Bonferroni test was used to compare treated groups to NX standard dose (0.1 mg/kg) (* *p* < 0.05; ** *p* < 0.01; *** *p* < 0.005). (**E**) Analgesia as a percentage of the maximum possible effect for all treated groups. The Bonferroni test was used to compare treated groups to NX standard dose (0.1 mg/kg) (* *p* < 0.05; ** *p* < 0.01; *** *p* < 0.005). (**F**) Rank order of OD reversal (mean time to all reflexes restored), HR AUC, RR AUC, and analgesia for all interventions. Error bars on the graphs denote standard error.

We analyzed the effectiveness of NX, NB and the combination to reverse respiratory depression by fentanyl in our model. We would like to point out that while the reduction in respiration was likely further impacted by muscle rigidity or apnea due to chest wall rigidity, airway obstruction [31] was not observed since the antidotes were effective nasally. Analysis of respiratory rates calculated as a percentage of resting rates (Figure 3A) showed that under most interventions, respiratory rates normalized within 6–8 min [25]. In contrast to that, 0.1 mg/kg of NB or NX as a monotherapy was the least effective intervention, leading to respiratory rate recovery only within 10–20 min.

With respiratory depression produced by usual therapeutic doses of NB being equivalent to that of morphine [34], one could expect an increased risk of hypoxic injury from a combination of NX + NB. To quantify a risk burden for the hypoxic injury, we examined RR AUC (RR × min) as a cumulative measure of respiratory activity (Figure 3B). To our surprise, we found that supplementation of NX with NB led to a reduction in the total severity of respiratory depression. Moreover, an NX + NB combination (0.1 + 0.025 mg/kg) worked significantly better that NX alone, increasing the respective RR AUC by about 1.6-fold. With no clear dose dependence, we concluded that the co-administration of NX with NB does not increase the risk of hypoxic injury but does provide a mild improvement in the respiratory rate.

In addition, as naloxone-induced withdrawal could be characterized by the activation of catecholaminergic neurons in the heart, we evaluated changes in the heart rate (HR) as a proxy for catecholamine surge. As expected, we found that HR in both naloxone-treated groups was elevated and stayed above the resting HR at all timepoints (Figure 3C). However, supplementation with NB completely mitigated this negative effect. When we looked at HR AUC (HR × min), a cumulative measure of heart rate, we found that compared to naloxone alone, all combinations resulted in statistically lower times for restoring HR (Figure 3D). These data suggest that κ-agonism could potentially mitigate the severity of the cardiorespiratory liabilities of naloxone [20].

### 2.3. Evaluation of Net Analgesia of NX, NB, and the Combination in Fentanyl-Induced Overdose Model

We evaluated the potential of various NX + NB combinations to address hyperalgesia and lower pain tolerance associated with opioid withdrawal and the need to self-medicate. We measured the latency of tail flick to thermal stimuli in rats before fentanyl administration (T-20 min) at baseline and 40 min after the intervention (Figure 2A). Figure 3E shows data for each treated group as a percentage of the maximum possible analgesia (%MPA). We found residual net analgesia (~5%) in NX-treated groups with an MPA of 100% (exceeding the 40 s predetermined cutoff) in NB-treated groups (Figure 3E). For all combinations, the increase in %MPA was NB dose dependent rather than ratio dependent. Also, for a combination of drugs, there was a threshold dose of NB (0.05 mg/kg) to produce a significant improvement in analgesia, as a combination with a lower dose of NB (0.025 mg/kg) had a %MPA similar to that of NX-only-treated animals.

Finally, we rank-ordered all interventions for overdose reversal potential, cumulative risks for cardiorespiratory liabilities, and analgesia with the lowest rank of 1 in the center of the graph (Figure 3F). All NX + NB combinations outranked all doses of NX only for the evaluated endpoints. Even combinations of a sub-therapeutic dose of NX (0.05 mg/kg) together with NB (0.05 mg/kg) outperformed the highest dose (0.2 mg/kg) of NX only. This finding indicates that with a combination therapy, not only can faster overdose reversal be achieved, but also higher doses of NX can be avoided to prevent the associated side effects.

## 3. Materials and Methods

### 3.1. Chemicals

Fentanyl (Fentanyl-Richter, 5 mL vial, 50 µg/mL, GEDEON RICHTER PLC, Budapest, Hungary) and naloxone (Forvel-Medochemie, Agios Athanassios, Limassol, Cyprus, 1 mL vial, 0.4 mg/mL) were purchased from commercial sources. Nalbuphine was used as the active substance, dissolved in 0.9% saline solution in a concentration of 0.8 mg/mL.

### 3.2. Fentanyl-Induced Overdose Model

Selection of optimal fentanyl dose: We used a single ascending dose study in 15 rats (*Rattus norvegicus*, Wistar; both genders) to determine the optimal fentanyl (Fentanyl-Richter, 5 mL vial, 50 µg/mL) dose. Starting from ED50 for rats (mentioned in the SPC of the product) of 0.013 mg/kg, five doses (0.013, 0.026, 0.052, 0.104, and 0.130 mg/kg) were tested on separate groups (1M + 2F or 2M + 1F) of rats. Monitoring 10 parameters, we selected 0.130 mg/kg of fentanyl for the study. To prevent death from cardiopulmonary arrest, naloxone (Forvel-Medochemie, 1 mL vial, 0.4 mg/mL) was administered immediately upon reaching an overdose.

Main study: In this study, a total of 20 rats (*Rattus norvegicus*, Wistar; both genders) were obtained from the laboratory animal facility—Centre for Experimental Medicine, University of Medicine and Pharmacy, Cluj-Napoca, Romania. The rats were 5–6 months old, and the body weight ranged from 201 to 415 g. The rats were acclimated for 1 week in a climate-controlled room maintained at 22 °C with approximately 60% relative humidity. Lighting was on a 12 h light/dark cycle, with food and water available ad libitum. Rats were randomized into 4 groups by body weight, each group consisting of 3M + 2F or 2M + 3F. Fentanyl was administered in a dose of 0.130 mg/kg intramuscularly while both naloxone and nalbuphine (with each active substance in powder form dissolved in 0.9% sterile saline solution in a concentration of 0.8 mg/mL) were administered intranasally. Individual vital and clinical sign monitoring (EDAN—IM8 VET system, San Diego, CA, USA) and evaluation were performed at all stages of the study before (ATp) and after (PTp) the administration of pharmacological substances.

Following fentanyl administration, the rats were monitored continuously during the first 2 min and then at 2 min intervals within the first 10 min and then at 10 min intervals for up to 60 min. Fentanyl overdose was considered to have occurred at the time of a significant reduction in respiratory (≤25% of the resting rate) and heart (≤80% of the resting rate) functions, with no corneal reflex detected. At that time, depending on the group tested, NB or its combination with NX was administered intranasally, marking the beginning of a time interval required for the complete recovery of the 10 monitored reflexes.

The intranasal administration was performed as follows. At the time of OD installation, the rat was monitored by equipment and was in sternal recumbency. The appropriate dose of antidote was prepared prior to fentanyl (FT) administration and drawn in a micropipette that allowed for exact dosing. At the time of administration, the rat’s head was raised at an angle of 25–35 degrees and the antidote was administered slowly over 20 s so that it did not reach the trachea or lungs, with 1/2 of the amount of antidote in each nostril.

### 3.3. Antinociception Evaluation

The determination of the antinociceptive effect was completed using the tail flick test [35,36] (TFT) with ethanol at −25 °C. The Neslab RTE 7 apparatus (ThermoElectron, Newington, CT, USA) with an adjustable temperature of between −25 °C and +150 °C (±0.01 °C) and 70% ethanol solution were used. The rats were placed in a transparent containment tube, with only the tail free. Half of the tail end was immersed in the cold 70% aqueous ethanol solution. The nociceptive threshold was taken as the latency until the rat flicked its tail from the bath. The time from immersion to tail flick was measured to the nearest hundredth of a second with a laboratory timer. To prevent tissue damage, a predetermined cutoff time of 40 s was used. No rats had frostbite or tail color change during the experiment. TFT was performed 20 min before fentanyl administration and again after 40 min. The maximum possible analgesia (%MPA) was calculated based on the following formula:

$$\%MPA=\left[\frac{Test-Pretest}{Cutoff-Pretest}\right]*100\%$$

All procedures included in the study complied with the guidelines of Directive 63/2010/EU and National Law 63/2014 on the protection of animals used for scientific purposes. The project was carried out with the approval of the Bioethics and Research Ethics Committee of the University of Agricultural Sciences and Veterinary Medicine Cluj-Napoca (no. 257/12 April 2021) and the project authorization issued by the National Sanitary Veterinary and Food Safety Authority (ANSVSA) no. 262/11 June 2021. The animals were accommodated and used for the experiments within the project in Unit for Breeding and Use of Laboratory Animals of the University of Agricultural Sciences and Veterinary Medicine Cluj-Napoca, which operates on the basis of the Veterinary Sanitary Authorization of the unit CJ 715 of 19 December 2016.

### 3.4. Statistical Analysis

Statistical analysis was performed using IBM SPSS Statistics for Windows 10, version 27.0 (IBMCorp, Armonk, NY, USA). One-way ANOVA with a Bonferroni post hoc test was used to compare differences of mean times to all reflexes restored since baseline across three treatment groups at each time interval. ANOVA was also used to compare HR changes in all treatment groups. The statistical significance level was set at α < 0.05. The synergism of the drug combination was evaluated according to the Loewe additivity model [37].

## 4. Conclusions

Further studies are needed with selective opioid agents, including κ-antagonists, to elucidate the precise mechanism of the observed phenomenon. However, our initial data strongly support the following conclusions.

Firstly, we have shown that supplementing naloxone with dual κ-agonist/μ-antagonist nalbuphine in a fentanyl-driven overdose model leads to significant improvements in full recovery and lower cardiorespiratory risks compared to animals treated with naloxone only.

Secondly, a distinct analgesic effect together with previously published reports on nalbuphine’s ability to reduce withdrawal severity and drug-seeking behavior, supplementing naloxone with nalbuphine, is likely to impact the need for self-medication and reduce the risk of post-discharge deaths.

Finally, as both medicines are commercially available, the development of combinations of them to reverse a fentanyl overdose should have the shortest path from this study to the bedside.

## Data Availability

The original contributions presented in the study are included in the article, further inquiries can be directed to the corresponding author/s.
